# High Arterial Blood-Pressure

**Published:** 1905-07-15

**Authors:** 


### Hospital Clinics.

### HIGH ARTERIAL BLOOD-PRESSURE.

There are few matters in which humanity is so little competent to avoid extremes as the matter of solicitude for personal health. Remote risks never command much consideration from a robust type of mind, and, indeed, it may truly be said that this capacious disregard of remote distresses is a criterion of the *mens sana in corpore sano.* Most healthy men of education, whatever their professed convictions, are not without a spark of fatalism, and it is no wonder that, in spite of the changes and chances of life daily enacted before their eyes, they decline to entertain warnings touching the distant future. Thus, by a somewhat bitter irony, the hypochondriac drags out a tortured existence well past its seventh decade, while the strenuous and cheerful, because careless, liver passes to his grave, regretful and regretted, in his fifth or sixth. There is, moreover, another point to be considered, namely, that while a large majority of men who at the age of fifty find themselves threatened with heart symptoms would gladly have a further twenty years added to their lives, and feel, perhaps, that any sacrifice in the past would have been well repaid by present health, yet most of us in the pride of health and strength view nothing with so much aversion as the possibility of a prolonged dotage. Therefore, in most instances, any argument which insists upon a deprivation of present indulgences—in themselves, perhaps, perfectly legitimate—for the avoidance of a problematical peril twenty years away, commonly falls upon deaf ears. None the less it is the duty of our profession to lay before our clients the means best calculated to maintain the highest state of health for the longest possible time, and thus to transfer the burden of responsibility to the shoulders of the sceptic or recalcitrant.

Much information has been collected as to the interdependence of high blood-pressure and arterial disease, but the exact relations of the two are not yet quite understood; there is no doubt that though arteriosclerosis and increased tension of the blood commonly go together, yet advanced arterial lesions are compatible, over long periods of time, with a blood-pressure in all respects normal. These latter cases are the exception, however, and Dr. Rolleston in a recent paper [1] has devoted himself to a consideration of the commoner variety of arteriosclerosis, namely, that depending upon heightened pressure in the arteries.

The causes which bring about this excessive intra-arterial tension by increasing the peripheral capillary resistance are tabulated by the author as follows:

1. Poisons taken into the body, whether inorganic, such as lead, or organic, such as alcohol or the complex waste products of proteid digestion. The last is the most important item in the case of sedentary men who habitually ingest large quantities of meat while the physical calls upon the body are progressively lessening as age advances.

2. Auto-intoxication by poisons manufactured in the body as the result of disordered function. This is the case in dyspepsia, in constipation, and in gout.

3. Imperfect excretion of poisons normally formed by the metabolic processes of the body, as is the case in parenchymatous disease of the kidney.

4. Nervous influences are capable of causing peripheral vaso-constriction and consequent high pressure in the arteries. Thus worry, and all the complex components of the latter-day "strenuous life" are potential sources of arteriosclerosis.

5. Excessive influence of the adrenal glands. It has been suggested that normally an equilibrium of blood-pressure is maintained by the balanced effects of thyroid and adrenal secretion, the former being vaso-dilator in function, the latter vaso-constrictor. Disturbance of this equilibrium will follow naturally upon loss of balance between the quantities of each secretion, and it is possible that the supra-renals, by an excessive activity, or the thyroid by a diminished one, may lie at the root of some cases of arteriosclerosis. It is significant, in this connection, that with myxœdema, a disease in which the thyroid secretion is notably deficient, arteriosclerosis is very common. Dr. W. Russell has further suggested that the proportions of these two secretions may be influenced by articles of diet, and that a copious diet of meat may thus bring about arteriosclerosis, either by stimulating the adrenals or by inhibiting the thyroid gland. This is, however, purely speculative; it is true that several observers have demonstrated hyperplasia of the adrenals in the subjects of granular kidneys, but not hyperplasia of the medullary parts of these bodies, in which alone resides the property of forming the constricting agent.

Speaking of the results of long-continued high-blood pressure, Dr. Rolleston points out that as the heart begins to fail before the excessive task allotted to it, the left ventricle dilates and the pulse becomes irregular; but in spite of this the arterial pressure may remain abnormally high. This is of great importance from the therapeutic point of view, for under such circumstances the administration of digitalis—the great resource for conditions of chronic heart-failure—may prove a fatal intervention, for this drug increases the already unanswered calls upon the heart-muscle by raising the blood-pressure, and may even cause the rupture of faulty arteries in the brain. The author has more than once seen hemiplegia follow this line of treatment in such patients.

Touching the recognition of high arterial tension, Dr. Rolleston advocates the use of some mechanical pressure-gauge as an assistance to the fingers. He recommends Stanton's modification of Riva-Rocci's instrument, which estimates the maximum systolic pressure in the artery. It is easy to apply and to read, and "controls the impressions gained by the finger, and educates the *tactus eruditus.*"

The treatment of high tension brings us back to the difficulties outlined above. It includes a departure from long-established habits of living; it demands moderation in diet (especially as regards the proteid elements), and absolute, or almost absolute, abstention from alcohol; exercise without excessive effort; care for the free action of the skin and all excretory apparatus; and, lastly, that greatest and most unattainable of blessings, the avoidance of worry, anxiety, and strain. Physicians may preach of these things, but, what with the urgent calls of temperament and the stereotyped custom of a lifetime, they cannot look for more than a superficial observance of their wise maxims. The man who submits himself to all these restrictions is thereby confessed an invalid, though his actual bodily discomforts at the time may be but trifling; and it may be questioned whether in a popular phrase "the game is worth the candle." The truth is, that in the matter of high blood-pressure we must, as Professor Allbutt has pointed out, treat processes and not the disease; that is to say, we must educate the young to habits of moderation, for it is idle to send a man to school when he is 50. With this, as with so many of the conditions which come within our purview, the whole truth is contained in the bull that "there is no cure but prevention."

[1] Clin. Jour., June 21, 1905.

